# Identifying inequitable healthcare in older people: systematic review of current research practice

**DOI:** 10.1186/s12939-017-0605-z

**Published:** 2017-07-11

**Authors:** Sarah M. Salway, Nick Payne, Melanie Rimmer, Stefanie Buckner, Hannah Jordan, Jean Adams, Kate Walters, Sarah L. Sowden, Lynne Forrest, Linda Sharp, Mira Hidajat, Martin White, Yoav Ben-Shlomo

**Affiliations:** 1 School of Health & Related Research, University of Sheffield, Regent Court, 30 Regent Street, Sheffield, S1 4DA UK; 2 Department of Public Health and Primary Care, University of Cambridge, Forvie Site, Robinson Way, Cambridge, CB2 0SR UK; 3 MRC Epidemiology Unit, University of Cambridge, School of Clinical Medicine, Cambridge, CB2 0QQ UK; 4 Centre for Ageing & Population Studies, Department of Primary Care & Population Health, University College London, Rowland Hill Street, London, NW3 2PF UK; 5 Institute of Health and Society, Newcastle University, Baddiley-Clark Building, Newcastle upon, Tyne NE2 4AX UK; 6 Administrative Data Research Centre, University of Edinburgh, Edinburgh Bioquarter, 9 Little France Road, Edinburgh, EH16 4UX UK; 7 School of Social and Community Medicine, University of Bristol, Canynge Hall, 39 Whatley Road, Bristol, BS8 2PS UK

**Keywords:** Equity, Disparity, Ageism, Methodology, Healthcare

## Abstract

**Background:**

There is growing consensus on the importance of identifying age-related inequities in the receipt of public health and healthcare interventions, but concerns regarding conceptual and methodological rigour in this area of research. Establishing age inequity in receipt requires evidence of a difference that is not an artefact of poor measurement of need or receipt; is not warranted on the grounds of patient preference or clinical safety; and is judged to be unfair.

**Method:**

A systematic, thematic literature review was undertaken with the objective of characterising recent research approaches. Studies were eligible if the population was in a country within the Organisation for Economic Co-operation and Development and analyses included an explicit focus on age-related patterns of healthcare receipt including those 60 years or older. A structured extraction template was applied. Extracted material was synthesised in thematic memos. A set of categorical codes were then defined and applied to produce summary counts across key dimensions. This process was iterative to allow reconciliation of discrepancies and ensure reliability.

**Results:**

Forty nine studies met the eligibility criteria. A wide variety of concepts, terms and methodologies were used across these studies. Thirty five studies employed multivariable techniques to produce adjusted receipt-need ratios, though few clearly articulated their rationale, indicating the need for great conceptual clarity. Eighteen studies made reference to patient preference as a relevant consideration, but just one incorporated any kind of adjustment for this factor. Twenty five studies discussed effectiveness among older adults, with fourteen raising the possibility of differential effectiveness, and one differential cost-effectiveness, by age. Just three studies made explicit reference to the ethical nature of healthcare resource allocation by age. While many authors presented suitably cautious conclusions, some appeared to over-stretch their findings concluding that observed differences were ‘inequitable’. Limitations include possible biases in the retrieved material due to inconsistent database indexing and a focus on OECD country populations and studies with English titles.

**Conclusions:**

Caution is needed among clinicians and other evidence-users in accepting claims of healthcare ‘ageism’ in some published papers. Principles for improved research practice are proposed.

**Electronic supplementary material:**

The online version of this article (doi:10.1186/s12939-017-0605-z) contains supplementary material, which is available to authorized users.

## Background

Inequity (or disparity) in the receipt of public health and healthcare interventions across social groups is of concern internationally [1–5]. Differences in the quality and quantity of healthcare received have been documented along a number of axes, particularly socioeconomic status [2, 3], gender [6], race/ethnicity [7], and geography [8], and across a range of public health and healthcare contexts. When such differences are labelled as ‘inequitable’ the implication is that they are unwarranted, unfair and avoidable. Differences in receipt by age are rightly receiving attention. Older people may be less likely to receive potentially beneficial treatment or interventions than younger people due to a range of factors [9, 10], resulting in poorer outcomes [11]. Given current trends towards ageing populations, it is unsurprising that a growing body of empirical studies [12, 13], commentary pieces [14–16] and policy documents [17] call for action on ‘age discrimination’ within health services and unmet healthcare need among older people.

However, while there appears to be growing consensus on the importance of identifying and tackling disparities in public health and healthcare receipt, concerns have been raised regarding the conceptual clarity and rigour of research in this field. The need for greater attention to measurement issues has been highlighted, particularly the importance of comparing levels of receipt in relation to established clinical need [18–20]. A more hypothesis-driven approach to comparisons with more careful consideration of selection biases and confounders has also been advocated [20–22]. Authors from disparate disciplines and specialties also argue for more consistent terminology and greater engagement with underlying debates [18]. In particular, the importance of ethical arguments relating to the potential trade-offs between addressing differential receipt on the one hand, and efficiency in resource allocation on the other, has been noted [23–27]. Further, the need to consider the multiple patient- and provider-side processes that might account for any observed differences in healthcare receipt has been emphasised, implying a need for caution in claims of ‘bias’ or ‘discrimination’ within health services [18]. Such critiques date back to the early 1980s and apply to work on age-related, as well as other, disparities. However, in relation to age, there has been no attempt to review practice or consolidate research principles to-date and it remains unclear whether research in this area has improved in recent years. Recognising that ‘state-of-the-art’ reviews can be valuable in steering researchers towards more rigorous and useful research practice [28, 29], we undertook a systematic, thematic literature review to characterise recent approaches to examining age-related differences in receipt of healthcare and public health interventions. The aims were to: (i) describe study framing, designs and methods, (ii) assess their strengths and weaknesses in relation to key methodological issues; and (iii) identify implications for future research practice.

## Methods

Below we follow the PRISMA guidelines [30] in reporting on the conduct of the review as far as they are applicable (and we note where it is appropriate to deviate from these in Additional file 1). A protocol was prepared for team use. The review was not prospectively registered.

### Search strategy

We adopted a systematic approach to identifying studies that quantitatively examined the receipt of a healthcare or public health intervention among older people. Initially, 73 key papers were recommended by experts and were used in a pearl-growing approach [31] to identify index terms and search vocabulary. Four databases (Cinahl, Psychinfo, Medline and Embase) were searched. We employed an iteratively developed set of MeSH and keyword terms combining synonyms referring to three fields: age-related factors; access; and healthcare/treatment [see Additional file 2 for an example]. The language restriction was that titles and abstracts were in English; the date restrictions were abstracts published January 1990-July 2014. Articles identified via this electronic search (*n* = 11,055) were combined with the key papers from experts, and those generated via citation searching and reference list checking to produce a total of 11,370 papers for sifting. Reference Manager Version 12 was used. A preliminary title sift was undertaken by two independent researchers, followed by an abstract review where necessary, to exclude papers clearly outside the scope of investigation. This resulted in 456 potential papers for inclusion.

### Study eligibility

Full texts of these 456 papers were read and included if they satisfied the following criteria: Population: based on a population from a country (ries) within the Organisation for Economic Co-operation and Development (OECD) and including people aged 60 years or over; Outcome: healthcare receipt (broadly defined including specific treatments or drugs, individually targeted public health interventions, as well as more general healthcare inputs such as treatment in a specialist stroke unit) Comparisons: an age-related comparative analysis of healthcare receipt in relation to health need; Study design: no restrictions. In addition, only studies that included an explicit focus on age-related patterns of healthcare receipt (as opposed to the analysis of receipt by age being a by-product of some other analytical focus) were eligible. The final inclusion criterion was important to ensure that the review focused on a body of studies that directly addressed the question of whether healthcare receipt differs by age and so could reasonably be synthesised to address our research aims. Recognising the subjective nature of this criterion, all papers were re-read by two researchers to confirm their eligibility against this criterion. Where the two researchers could not agree, a third team member was consulted to reach consensus on inclusion/exclusion. Since our aim was to examine the state of recent research in this area, no quality-related inclusion or exclusion criteria were used.

### Extraction and synthesis

We undertook a directed (deductive) content analysis of the included studies. That is, we employed a structured systematic coding approach to classify parts of the text of the included studies based on the key conceptual and methodological themes already identified from the wider healthcare disparities literature cited above [32, 33]. An extraction template was drafted, piloted by four researchers, refined and finalised before being applied to all included papers. The final template included 55 extraction fields (including both open-ended responses and closed, categorical codes) across four aggregate themes: conceptual framing and rationale; sources of data and measures; analytical approaches to describing age-related differences; and establishing inequity (see Additional file 3 *for details of the extraction fields*). A template guide was also prepared to provide descriptors against each of the more interpretive extraction fields to support consistency of extraction across the research team. Data extraction was undertaken by four researchers, each of whom extracted a sub-set of papers across the whole template, with validation by a second researcher for around one quarter of coded extractions. Papers were read and re-read and material extracted into the template codes in three ways: cut-and-paste of verbatim excerpts (for simple descriptors such as date and key terms, definitions, arguments and numerical results); paraphrasing and precis of longer textual passages; or selection of the relevant code for categorical fields. Page and line numbers of extracted material were recorded to facilitate review. Extracted material from all studies was then synthesised by one researcher for each of the four thematic areas. Draft memos including both quantitative and thematic summaries were prepared and discussed by the four researchers. A set of categorical codes were then defined and applied to the extracted textual data to enable summary counts to be reported across the following key dimensions of study methodology:

- Contra-indications discussed (yes/no); contra-indications adjusted for in analyses (yes/no)
- Patient preferences discussed (yes/no); patient preferences adjusted for in analyses (yes/no)
- Confounding factors included in multivariable analyses (yes/no); adjustment for confounders explained and justified (yes/no)
- Treatment effectiveness at older age discussed (yes/no); differential effectiveness considered in the analyses (yes/no)
- Ethical/moral arguments surrounding healthcare allocation by age discussed (yes/no)
- Conclusion drawn regarding differential receipt (unwarranted difference suggested; unwarranted difference concluded; warranted difference suggested; warranted difference concluded; no conclusion made beyond report of age-related patterns)

This process was iterative, with original articles being revisited as often as necessary to reconcile discrepancies and ensure reliability.

## Results

### Study designs and characteristics

**Fig. 1:**
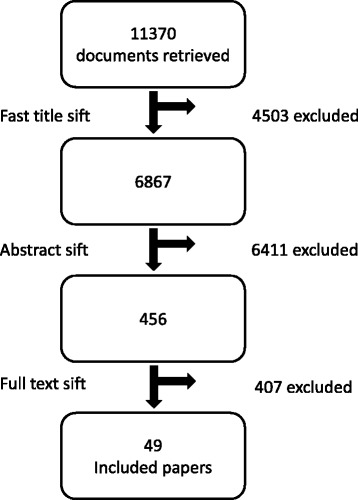
Flow diagram of search and sift process

Forty nine papers were included (see Fig. 1) from the 456 abstracts that were reviewed. Twenty studies referred to USA or Canada, 21 to European countries and 8 to other OECD countries. All papers were published after 2001; almost two thirds after 2010. A wide range of specialities and conditions were covered including musculoskeletal disease, epilepsy, oral health and asthma. Cardiovascular disease and cancer were equally represented and together made up the focus of half the studies. Mental health was addressed in eight studies *(see* Additional file 4 *for key descriptors for all included studies)*. Thirty studies examined patterns of receipt across all adult age groups, while 19 compared younger and older “elderly” groups. Nineteen studies adopted a cross-sectional design, and 30 were longitudinal. Measuring need in studies of potential healthcare disparities requires careful conceptualisation and standardized measurement [20]. Need was rarely defined in the studies, instead it was usually implied as capacity to benefit from care, or from a particular intervention. Data sources were most often: multi-centre research databases (including case-registries) (*n* = 23); routine healthcare system databases (*n* = 12); and single-centre research databases (*n* = 6). These sources generally provided large samples, with the median number of individuals with identified need being around 8,000. Methods of establishing need were varied including: clinical diagnosis by a healthcare professional (*n* = 27, usually recorded within a routine healthcare database); non-clinical, structured assessment using a tool (*n* = 14, either self-completion or administered during a cross-sectional survey); self-reports of symptoms, existing disease state or health issue requiring care (*n* = 4, usually reported in a cross-sectional survey). Other less common examples of need measurement included: death (with retrospective examination of use of healthcare); laboratory data diagnosis; and modelling demographic and related morbidity data from a study of a similar population to provide a proxy for incidence/prevalence (and hence need) in the study group; imputation in a population from measurement of incidence/prevalence in a similar group. Around two-thirds of the measures were validated or defined. Five studies considered that the measure of need might perform differently across age-groups, though analyses did not account for this. In 10 studies, authors attempted to provide a more refined measure of need by including measures of disease stage or severity within multivariable analyses. For example, Sin and Tu examined receipt of inhaled steroid therapy in asthma patients and included measures of disease severity in their multivariable models [34]. Twenty two studies examined receipt of specific treatments or interventions, 23 studies explored receipt of care defined more broadly (e.g. Reuber et al. examined receipt of care from a specialist epilepsy nurse [35]), and four included both specific and more general measures of receipt. Measures of receipt came from multi-centre research databases (*n* = 21); routine healthcare system databases (*n* = 14); single-centre research databases (*n* = 7) and other sources such as a national survey (*n* = 6), with one study not specifying the source.

### Conceptual framing and rationale

The study rationale and focus of the papers varied. Twenty three papers were framed solely in terms of the importance of exploring differential healthcare receipt between age-groups. Six papers were framed in terms of adherence to clinical guidelines or quality of care. Four were framed in terms of unmet need for healthcare within older age-groups, without reference to younger people. Multiple framing was found in 16 papers, most combining quality/guideline-adherence and differences between age-groups. A diversity of nomenclature was used in relation to age-related patterns of healthcare receipt (Table 1) with few explicit definitions. Twelve papers used the term ‘inequity’ (or equity or their derivatives) and 13 papers employed the term ‘disparity’ (more commonly used in USA), though only four gave definitions, all relating to ‘equity’. Forty one papers included some consideration of the factors that might shape healthcare access among older people, often referring to the attitudes or behaviours of healthcare practitioners. However, in the main this was confined to the background or discussion sections of the papers. Just nine studies referred explicitly to a conceptual framework that guided the analysis. For example, Litwin and Sapir [36] made reference to the Andersen-Newman model of health service utilisation [37] and Park [38] drew on the Aday and Andersen access framework [39].

**Table 1:** Terms used to refer to age-related differential healthcare delivery or receipt

Age bias	Foregone care	Withholding effective treatment
Age-related	Gaps	Overlooked group
Age-dependent	Guideline deviation	Over-use
Ageism	Incomplete diagnostic assessment	Poorer access to appropriate care
Ageist in terms of access to provision	Inequality/inequalities	Poor quality of care
Ageist neglect	Inequity/inequitable	Relinquishment of care
Barriers to care	(In) appropriate care	Reluctance
Decreased use	Less likely to receive	Restriction in access
Denial of treatment	Limited use	Relative neglect
Difference/differences	Lower use	Risk-treatment paradox
Disparity/disparities	Low levels of receipt	Structural inequalities
Differential/differentials	Misfit between care and needs	Sub-optimal management
Disadvantaged	Not receiving adequate investigations and community therapy	Treated less aggressively
Discrimination	Universal access	Under-treatment
Disproportionate	Unmet need	Under-utilisation/under-use
Dissimilarities in care/utilisation	Variations	Under-representation
Discrepancy	Variability	Unfair system
Denied access	Variations based on non-clinical factors	Uneven distribution
Equity in access		
Equity/equitable		

### Study strengths and weaknesses in relation to key methodological issues

#### Analytical approaches to describing age-related differences

Among the 49 studies, 21 reported findings related to more than one receipt-need outcome. Thirty six reported that younger people with the relevant healthcare need were more likely to have received a treatment/service than older people with the same need (although in six of these studies the findings were not in this direction for every outcome studied). Seven studies reported that older people with the relevant healthcare need were more likely to have received a treatment/service than younger people with the same need. Nine studies reported no difference in use-need ratios between older and younger age groups for one or more of the treatments/services studied.

Explorations of differential health-care receipt must carefully consider confounding factors and sources of clinically warranted variation, necessitating sophisticated analyses. We found that 14 studies employed only unadjusted analyses, calculating the proportion of patients identified with need that received the treatment/service in question, and compared these receipt-need ratios across age (for example Jin et al. [40] reported the percentage of Korean patients diagnosed with rheumatoid arthritis who were prescribed disease-modifying antirheumatic drugs (DMARDs) by age-group as follows: 65–69 years (15.8%), 70–74 years (12.2%), 75–79 years (9.0%) and 80+ years (5.0%)). By contrast, 35 studies employed multivariable techniques to produce adjusted receipt-need ratios. There was no evidence of change over time, with 78% and 68% of papers published before and since 2010 using adjusted analyses respectively. Adjusted analyses suggest an awareness of the need to rule out appropriate variation, though the rationale for inclusion of variables in adjusted analyses was rarely clearly articulated.

Older people are more likely than younger people to have comorbidities and to be receiving other treatments, raising the possibility that non-receipt of treatment is appropriate due to potential harm. Twenty six studies made no explicit reference to such contra-indications. In 18 of these, consideration of contra-indications could be considered not relevant, since they examined access to generic healthcare services (for example Burge et al. examined access to palliative care among cancer patients [41]). However, in eight other studies, this omission makes interpretation problematic as non-receipt may or may not have been appropriate (for example Situmorang et al. examined treatment for prostate cancer [42]). Thirteen studies included measures of comorbidity within their statistical analyses, but in eight of these no clear justification for their inclusion was provided.

#### Patient preferences

Patient preference should also be considered in analyses of comparative healthcare receipt since systematic differences in informed decision-making between age-groups could result in appropriate variation in receipt [21]. Eighteen studies made reference to this consideration, for example, Bhalla et al. note: *“When considering the uptake of carotid Doppler and brain imaging, this may have been appropriately withheld from some older patients according to their wishes*”([43], p622). Just one study, by Hunt et al. [44], employed a measure of patient preference in their main analysis of receipt, by examining whether patient preference for place of death was achieved, along with other measures of the type of palliative care received. Three other studies examined this issue to some extent. Bajorek and Ren [45] and Hermosillo-Rodriguez et al. [46] present some univariate data on patient refusal as a reason for non-receipt. Palmcrantz et al. [47] present an interesting analysis that suggests there are age-related differences in expectations of recovery following stroke since disparities in the provision of health care between younger and older patients were not reflected in differences in self-perceived global recovery after 1 year.

#### Differential effectiveness

A further factor that might be considered as justification for age variation in receipt-use ratios is differential effectiveness, and cost effectiveness, by age, of the treatment/care under investigation. Eighteen of the studies included no explicit consideration of effectiveness of the treatment/care being studied; in most cases general services rather than specific treatments. Six further studies made general statements regarding treatment effectiveness or cited general clinical guidelines, without specific reference to effectiveness at older ages.

In contrast, 25 studies did discuss effectiveness with respect to age. Twelve of these referred to evidence-based guidelines, or prior research findings, that supported effectiveness of the treatment in older age-groups. For example, Prina et al. state *“Psychological interventions have been used successfully for the treatment of anxiety disorders in older age, as demonstrated by the large number of meta-analyses that have shown the benefits of interventions over control conditions. Psychological therapies have also been shown to be effective in the treatment of depression in later life”* ([48]; p75).

Fourteen studies raised the possibility of differential effectiveness by age, though in contrasting ways. Some papers identified the possibility of age-related differences in risk reduction with treatment, for instance physiological differences in responsiveness to drugs, so that the direct benefit among treated individuals could be lower among older age-groups than younger ones. In contrast, two studies noted that the baseline risk of adverse outcome in those identified with need is likely to be higher in older age-groups so that, if treatment reduces this risk to the same extent in all age-groups, the numbers needed to treat (NNT) statistic would be lower in older age-groups than younger age-groups. Both of these lines of argument relate to the possibility of age-related differences in absolute risk reduction (i.e. direct benefit). In other studies, more general concepts of differential effectiveness were invoked, as authors referred to differences in rates of co-morbidity and lower survival chances among older age-groups. Several such studies suggested that lower receipt of the treatment/care under consideration might be partially or wholly warranted if this reflected a clinical judgement of insufficient future benefit. For example, Bhalla et al. state “*Therapy input for older patients, however, may have been quite appropriately withheld from patients who survived in a very poor functional state on stroke units, and resources diverted to younger patients where the need was perceived to be greater”* ([43], p620).

However, as noted above, while 13 studies produced receipt-need ratios adjusted for measures of co-morbidity, few explicated the logic behind such adjustment (i.e. whether this was intended to rule out warranted variation due to contra-indications or adjust for differential future life expectancy). Furthermore, just one study made explicit mention of the possibility of age-related differences in cost effectiveness, stating that non-treatment in the elderly “could not be rationalized on life expectancy or cost effectiveness arguments” ([49] p4328).

#### Establishing inequity

Having highlighted some potential limitations in many of the papers reviewed, it is important to note that unavailability of some key variables will often limit studies to simple descriptive analyses. As noted above, few studies explicitly used the terms ‘inequity’ or ‘disparity’, and many authors acknowledged limitations of the analyses undertaken. Nevertheless, review of the conclusions drawn found that some papers went beyond simply reporting observed differences. Table 2 summarises the conclusions drawn, and the factors that were discussed and taken into account in the analyses performed, for those 36 studies that reported higher healthcare receipt among younger than older people for one or more outcomes. We identified that 27 papers concluded or suggested that the difference found was unwarranted *(see* Additional file 5 *for more detail on the extracted information and coding)*. Among the fifteen papers that concluded evidence of an unwarranted ‘pro-younger’ differential in healthcare receipt, seven made no reference to possible contraindications and 11 no reference to patient preferences. Twelve of these papers suggested that practitioner behaviours and/or health system factors were possible explanations for the observed differences. Regardless of the conclusion drawn, very few papers made any reference to the ethical nature of judgements regarding healthcare resource allocation by age, just one paper included any kind of adjustment for patient preference and none included any adjustment for differential effectiveness (or cost effectiveness). There was no clear indication that the more cautious papers were more recently published.

**Table 2:** Summary of findings reported, factors considered and conclusions drawn among those studies reporting higher receipt among younger than older groups (*N* = 36)

	Unwarranted difference concluded	Unwarranted difference suggested	Warranted difference suggested	No conclusion beyond report of difference
Contraindications				
Not mentioned	7	5	0	3
Discussed only^a^	1	4	1	4
Adjusted for	7	3	0	1
Patient preference				
Not mentioned	11	7	0	3
Discussed only^a^	4	4	1	5
Adjusted for	0	1	0	0
Effectiveness at older age				
Not mentioned	3	6	0	2
Discussed only^a^	12	7	1	6
Adjusted for	0	0	0	0
Ethical nature of judgement acknowledged	0	0	0	2
Total number of papers	15	12	1	8

^a^Some studies discussed a possible explanatory factor without also performing an associated analysis either because the data were not available and/or because authors did not consider the factor to be justification for differential receipt

## Discussion

This review addressed the significant interest in exploring patterns of healthcare receipt by age and persistent concerns regarding ‘inequitable’ access for older people evident across research and policy [14–17]. The review found a wide variety of concepts, terms and methodologies, and some important shortcomings in some published studies. Establishing the existence of age inequity in the receipt of healthcare requires evidence of a difference that is not an artefact of poor measures of need or receipt and is not warranted on the grounds of patient preference, clinical safety or cost-effectiveness. It also requires an ethical judgement that the observed difference is unfair. Our review found that most studies engaged with some but not all of these considerations.

Fewer than half the studies acknowledged that patient preference should be taken into account, and one study incorporated this factor into analyses. This is a complex area, however, since even if patient preferences are recorded, such choices may be inadequately informed and/or made on the basis of wider circumstances that could be considered inequitable [21, 50]. For instance, older patients may refuse certain treatments because they have insufficient social support to get through the recovery period. In terms of clinically appropriate variation, most studies showed awareness of the importance of ruling out contra-indications, but many failed to employ suitable analytical procedures, often because required data were unavailable. There is clearly often a trade-off between the large sample sizes but limited variables that are available in routine datasets.

Studies also varied in terms of whether and how potential age-differences in the clinical effectiveness of interventions were considered. It could be argued that studies that examined adherence to national clinical guidelines effectively side-stepped the need to explicitly consider this dimension. Furthermore, the lack of data from trials of effectiveness at older ages is an important obstacle in many treatment areas [51]. This requires a judgement as to whether, in the absence of evidence, not providing treatment is appropriate or that older patients should be treated as a default position, unless there are contra-indications (which may be more common in older patients). Nevertheless, the large number of studies that controlled for measures of co-morbidity (as well as other variables) within multivariable regression models without a clearly articulated rationale indicates the need for greater conceptual transparency (an issue that has been pointed out more generally by Pocock et al. [52]).

Several studies referred to the lower survival chances of older individuals as an explanation (and potential justification) for lower healthcare receipt, without making any reference to the associated moral arguments. Clinical decision-making on the basis of patient sub-grouping, and the associated potential benefits and harms, does happen in practice, and population-level assessments of healthcare cost-effectiveness are often made on the basis of years of healthy life to be gained (e.g. UK NICE [53]). However, whether or not such resource allocation decisions are considered appropriate is a moral, as well as a technical, judgement [54, 55].

It should be noted that studies varied in their framing with some having a stronger focus on examining (in)equity than others, and that study designs were sometimes necessarily constrained by data availability. Nevertheless, while some studies acknowledged the limitations of analyses performed and presented suitably cautious conclusions, others appeared to over-stretch their findings concluding that the differences observed were unwarranted.

### Strengths and limitations

The present study faced challenges in searching for relevant literature within electronic databases. Inconsistent indexing meant that relevant material is likely to have been overlooked, and it is possible that we would find different patterns of research practice if alternative search strategies had been employed. In particular, using key word synonyms related to healthcare/treatment resulted in relatively few studies exploring receipt of public health interventions by age. Further, restricting our focus to OECD country populations and studies with English titles may have introduced bias, though it is not possible to speculate on its nature. Nevertheless, our systematic approach resulted in 49 recent studies across a wide range of specialties and settings from which we have identified several characteristics of current methodological approaches that warrant attention.

### Implications for practice and future research

Our findings suggest caution on the part of clinicians and other evidence-users in accepting the claims of public health or healthcare ‘ageism’ found in some published papers. They also challenge researchers to improve their research practice and reporting of findings and invite a much more explicit engagement with the complexity of establishing inequitable patterns of care receipt. Table 3 suggests some principles for good practice in this area of research, which we hope will help future studies.

**Table 3:** Principles for research on age-related inequalities in healthcare receipt

• Use consistent terminology and provide definitions for key terms
• Use a theoretical framework to guide analyses that clearly articulates hypothesised relationships between age, mediating mechanisms, moderating factors and receipt of healthcare
• Adjust for need using measures that are validated across age and incorporate severity where appropriate
• Account for co-morbidities that might preclude treatment (contra-indications) or reduce the likelihood of receiving interventions and that may affect assessment of the benefit-to-harm ratio
• Carefully consider patient preferences and adjust for these wherever feasible
• Consider differential clinical effectiveness and cost effectiveness by age, including both the capacity to benefit and the risk of harm
• Explicitly acknowledge the inherent moral dimensions of resource allocation across ages
• Clearly articulate study limitations and exercise caution in concluding equitable or inequitable patterns of care

This study examined how researchers have approached the question of whether there is equal receipt of specific treatments/interventions/services for equal need across ages. How researchers have tackled other equity-related issues warrants attention in future, such as: whether similar needs are met via different healthcare (or other) inputs at different ages; whether the processes and experiences of accessing care, including dimensions of quality, differ by age; and indeed whether and how equity concerns shape the development of interventions and treatments [56]. Future research should also explore how the interplay of multiple axes of disadvantage – for instance, race/ethnicity, gender, socioeconomic status and age – are being addressed within healthcare disparities research. Research is also needed to generate better understanding of the patient-, provider- and system- related factors that generate observed patterns of healthcare receipt by age so that action to address established inequities, at all levels, can be developed.

## Conclusion

There is growing research interest in documenting patterns of public health and healthcare receipt by age and in identifying instances of inequity for older people. Currently, conclusions are often compromised by data limitations and/or a lack of conceptual and methodological rigour. The variability in approach across the studies reviewed suggests opportunities for researchers to share good practice.

## Additional files

Additional file 1: Search strategy description (DOC 62 kb)

Additional file 2: PRISMA checklist (DOCX 13 kb)

Additional file 3: Description of extraction codes (DOCX 17 kb)

Additional file 4: Description of included studies (DOCX 31 kb)

Additional file 5: Studies reporting higher receipt-need ratio among younger than older groups in at least one outcome (*N* = 36) (DOCX 26 kb)
