# Supplementary material for: Identifying inequitable healthcare in older people: systematic review of current research practice
Source: Int J Equity Health. 2017 Jul 11;16:123. doi: 10.1186/s12939-017-0605-z (PMC5505033; doi:10.1186/s12939-017-0605-z)
Supplement: Supplementary file 2 — PRISMA checklist (DOCX 13 kb) [file 12939_2017_605_MOESM2_ESM.docx]

**Additional Table 4: Search strategy**

Search strategy for all databases: Cinahl, Psychinfo, Medline, Embase.

| 1 | age factor*.ti,ab,sh. |
| --- | --- |
| 2 | (ageing or old person or old people or older person or older people or elderly).ti,ab. |
| 3 | age related.ti,ab. |
| 4 | age specific.ti,ab. |
| 5 | age proof*.ti,ab. |
| 6 | or/1-5 |
| 7 | (access* or utilis* or utiliz* or inequalit* or inequit* or uptake or usage or unequal).ti,ab. |
| 8 | 6 and 7 |
| 9 | (service* or treatment* or health care or healthcare).ti,ab. |
| 10 | 8 and 9 |
| 11 | limit 10 to (yr="1990 -Current" and english) |
