# Supplementary material for: Identifying inequitable healthcare in older people: systematic review of current research practice
Source: Int J Equity Health. 2017 Jul 11;16:123. doi: 10.1186/s12939-017-0605-z (PMC5505033; doi:10.1186/s12939-017-0605-z)
Supplement: Supplementary file 3 — Description of extraction codes (DOCX 17 kb) [file 12939_2017_605_MOESM3_ESM.docx]

**Additional File 3: Extraction fields within thematic areas**

| **Basic Study Descriptors** | **Use-Need Relationship Analysis** | **Framing and Conceptualisation**  (all free text verbatim or precis) | **Use-Need Relationship (Advanced)**  (all free text verbatim or precis) |
| --- | --- | --- | --- |
| Title | Total number | Framing of the paper | Frailty |
| 1st Author | Number with Need | Framing of the paper - conceptual categories | Polypharmacy |
| Date | Measure of Need (free text) | Terms and definitions related to equity (outcomes) | Co-Morbidity |
| Country | Measure of Need (categorical) | Theory on access (General) | Differential Effectiveness |
| Journal & volume/page | Is need measure validated? | Theory on differential/ Inequitable access | Other axes of difference |
| Other authors | Data source for need (free text) | Terms and defns - (in)equity generating processes | Sub-groups/ Intersectionality |
| Specialty | Data source for need (categorical) | Establishing inequity – effectiveness | Explanatory factors |
| Specialty Category | Measure of use (free text) | Establishing inequity - benefits versus risks | Other comments on advanced analysis |
| Condition | Measure of use (categorical) | Establishing inequity - contra-indications | Inequity-related conclusions |
| Condition Category | Data source for use (free text) | Conclusions | Comments |
| Study Design | Data source for use (categorical) | Implications |  |
|  | Analysis (Free text) | Economic arguments |  |
|  | Analysis (Categorical) | Interventional strategies |  |
|  | Results (free text) | Any other comments on conceptual dimensions |  |
|  | Results (categorical) |  |  |
|  | Summary statistic |  |  |
|  | Age comparisons (free text) |  |  |
|  | Age comparisons (categorical) |  |  |
|  | Stated Limitations |  |  |
|  | Other design limitations |  |  |
