# Supplementary material for: Identifying inequitable healthcare in older people: systematic review of current research practice
Source: Int J Equity Health. 2017 Jul 11;16:123. doi: 10.1186/s12939-017-0605-z (PMC5505033; doi:10.1186/s12939-017-0605-z)
Supplement: Supplementary file 4 — Description of included studies (DOCX 31 kb) [file 12939_2017_605_MOESM4_ESM.docx]

**Additional File 4: Description of Included Studies (n=49)**

| # | Title | Authors | Date | Journal | Country | Condition | Study Design |
| --- | --- | --- | --- | --- | --- | --- | --- |
| 1 | Utilization patterns of disease-modifying antirheumatic drugs in elderly rheumatoid arthritis patients | Jin X, Lee J, Choi N, Seong J, Shin J, Kim Y, Kim M, Yang B, Park B | 2014 | Journal of Korean Medical Science. 2014 Feb 1;29(2):210-6. | Korea | Rheumatoid arthritis | Cross sectional |
| 2 | The effect of age and comorbidity on patient-centered health outcomes in patients receiving adjuvant chemotherapy for colon cancer | Hermosillo-Rodriguez J, Anaya D, Sada Y, Walder A,  Amspoker A, Berger D, Naik A | 2013 | Journal of Geriatric Oncology. 2013 Apr 30;4(2):99-106. | USA | Colon cancer | Prospective |
| 3 | Age differences in the adherence to treatment guidelines and outcome in patients with ST-elevation myocardial infarction | Gottlieb S, Behar S, Schwartz R, Harpaz D, Shotan A, Zahger D, Hod H, Tzivoni D, Moriel M | 2011 | Archives of Gerontology and Geriatrics. 2011 Feb 28;52(1):118-24. | Israel | MI | Prospective |
| 4 | Do older adults have equitable access to specialist epilepsy services? | Reuber M, Torane P, Mack C | 2010 | Epilepsia. 2010 Nov 1;51(11):2341-3. | UK | Epilepsy | Cross sectional |
| 5 | Influence of age on the management of heart failure: Findings from Get With the Guidelines–Heart Failure (GWTG-HF) | Forman D, Cannon C, Hernandez A, Liang L,  Yancy C, Fonarow G | 2009 | American Heart Journal. 2009 Jun 30;157(6):1010-7. | USA | Heart failure | Retrospective |
| 6 | Rectal cancer treatment and outcome in the elderly: an audit based on the Swedish rectal cancer registry 1995–2004 | Jung B, Påhlman L, Johansson R, Nilsson E | 2009 | BMC Cancer. 2009 Feb 26;9(1):1. | Sweden | Rectal cancer | Prospective |
| 7 | A population-based study of age inequalities in access to palliative care among cancer patients | Burge F, Lawson B, Johnston G, Grunfeld E | 2008 | Medical Care. 2008 Dec;46(12):1203. | Canada | Cancer | Retrospective |
| 8 | Mental health service use among older African Americans: The national survey of American life | Neighbors H, Woodward A, Bullard K, Ford B, Taylor R, Jackson J | 2008 | The American Journal of Geriatric Psychiatry. 2008 Dec 31;16(12):948-56. | USA | Any mental health condition | Cross sectional |
| 9 | Use of thrombolytic therapy for acute myocardial infarction: Effects of gender and age on treatment rates | Kaplan K, Fitzpatrick P, Cox C, Shammas N, Marder V | 2002 | Journal of Thrombosis and Thrombolysis. 2002 Feb 1;13(1):21-6. | USA | MI | Retrospective |
| 10 | Improving access to psychological therapies and older people: Findings from the Eastern Region | Prina A, Marioni R, Hammond G, Jones P, Brayne C, Dening T | 2014 | Behaviour Research and Therapy. 2014 May 31;56:75-81. | France | Mental health care needs | Retrospective |
| 11 | Ageing and the prevalence and treatment of mental health problems | Jokela M, Batty G, Kivimäki M | 2013 | Psychological Medicine. 2013 Oct 1;43(10):2037-45. | UK | Mental health care needs | Prospective |
| 12 | Utilization of mental health care services among older adults with depression | Crabb R, Hunsley J | 2009 | Journal of clinical Psychology. 2006 Mar 1;62(3):299-312. | Canada | Depression | Cross sectional |
| 13 | Access to general practitioner services: The disabled elderly lag behind in underserved areas | Chaix B, Veugelers P , Boëlle P, Chauvin P | 2005 | The European Journal of Public Health. 2005 Jun 1;15(3):282-7. | France | All conditions in respect of Primary Care Services | Retrospective |
| 14 | Factors related to underuse of surveillance mammography among breast cancer survivors | Keating N, Landrum M, Guadagnoli E, Winer E, Ayanian J | 2006 | Journal of Clinical Oncology. 2006 Jan 1;24(1):85-94. | USA | Breast cancer | Retrospective |
| 15 | Older stroke patients in Europe: stroke care and determinants of outcome | Bhalla A, Grieve R, Tilling K, Rudd A, Wolfe C | 2004 | Age and Ageing. 2004 Nov 1;33(6):618-24. | UK | Stroke | Prospective |
| 16 | End-of-life care and preferences for place of death among the oldest old: Results of a population-based survey using VOICES–Short Form | Hunt K, Shlomo N, Addington-Hall J | 2014 | Journal of Palliative Medicine. 2014 Feb 1;17(2):176-82. | UK | Non-specific | Cross sectional |
| 17 | Factors affecting unmet healthcare needs of older people in Korea | Ahn Y, Kim N, Kim C, Ham O | 2013 | International Nursing Review. 2013 Dec 1;60(4):510-9. | Korea | Any healthcare needs | Cross sectional |
| 18 | Treatment of breast cancer in the elderly: A prospective, population-based Swiss study | Joerger M, Thürlimann B, Savidan A, Frick H, Rageth C, Lütolf U, Vlastos G, Bouchardy C, Konzelmanni I, Bordoni A, Probst-Hensch N, Jundtn G, Essa S | 2013 | Journal of Geriatric Oncology. 2013 Jan 31;4(1):39-47. | Switzerland | Breast cancer | Prospective |
| 19 | The impact of age on colorectal cancer incidence, treatment and outcomes in an equal-access health care system | Steele S, Park G, Johnson E, Martin M, Stojadinovic A, Maykel J, Causey M | 2014 | Diseases of the Colon & Rectum. 2014 Mar 1;57(3):303-10. | USA | Colorectal cancer | Retrospective |
| 20 | Age-dependent care and long-term (20 year) mortality of 14,434 myocardial infarction patients: Changes from 1985 to 2008 | Nauta S, Deckers J, Akkerhuis K, van Domburg R | 2013 | International Journal of Cardiology. 2013 Aug 10;167(3):693-7. | Netherlands | MCI | Retrospective |
| 21 | Age bias in physicians' recommendations for physical activity: A behavioral model of healthcare utilization for adults with arthritis | Austin S, Qu H, Shewchuk R | 2013 | Journal of Physical Activity & Health. 2013 Feb 1;10(2):222-31. | USA | Arthritis | Cross sectional |
| 22 | Equity in the use of publicly subsidized psychotherapy among elderly Danish cancer patients – a register-based cohort study | Von Heymann-Horan A, Bidstrup P,  Kristiansen L, Olsen A, Andersen K,  Elsass P, Johansen C, Dalton S | 2013 | Acta Oncologica. 2013 Feb 1;52(2):355-63. | Denmark | Cancer | Prospective |
| 23 | Treatment of hypertension in the elderly: Data from an international cohort of hypertensives treated by cardiologists | Thoenes M, Spirk D, Böhm M, Mahfoud F, Thevasan J, Bramlage P | 2013 | Journal of Human Hypertension. 2013 Feb 1;27(2):131-7. | Germany | Hypertension | Cross sectional |
| 24 | Prostate cancer in younger and older patients: Do we treat them differently? | Situmorang G, Umbas R, Mochtar C,  Santoso R | 2012 | Asian Pacific Journal of Cancer Prevention. 2012;13(9):4577-80. | Indonesia | Prostate cancer | Cross sectional |
| 25 | Differences between younger and older individuals in their use of care and rehabilitation but not in self-perceived global recovery 1 year after stroke | Palmcrantz S, Holmqvist L, Sommerfeld D, Tistad M,  Ytterberg C, von Koch L | 2012 | Journal of the Neurological Sciences. 2012 Oct 15;321(1):29-34. | Sweden | Stroke | Prospective |
| 26 | Utilisation of antithrombotic therapy for stroke prevention in atrial fibrillation in a Sydney hospital: Then and now | Bajorek B, Ren S | 2012 | International Journal of Clinical Pharmacy. 2012 Feb 1;34(1):88-97. | Australia | Any condition indicative for antithrombotic use | Retrospective |
| 27 | Disparities in trauma center access of older injured motor vehicular crash occupants | Ryb G, and Dischinger P | 2011 | Journal of Trauma-Injury, Infection, and Critical Care. 2011 Sep 1;71(3):742-7. | USA | Motor vehicle crash occupants | Cross sectional |
| 28 | Evaluation of antithrombotic usage for atrial fibrillation in aged care facilities | Singh P, Arrevad P, Peterson G, Bereznicki L | 2011 | Journal of Clinical Pharmacy and Therapeutics. 2011 Apr 1;36(2):166-71. | Denmark | Atrial fibrillation | Retrospective |
| 29 | The treatment of common mental disorders across age groups: Results from the 2007 adult psychiatric morbidity survey | Cooper C, Bebbington P, McManus S, Meltzer H, Stewart R, Farrell M, King M, Jenkins R, Livingston G | 2010 | Journal of Affective Disorders. 2010 Dec 31;127(1):96-101. | UK | Any psychiatric illness | Cross sectional |
| 30 | Chronic low back pain among older adults: A population-based perspective | Knauer S, Freburger J, Carey T | 2010 | Journal of Aging and Health. 2010 Jul 22(8) 1213–1234. | USA | Chronic low back pain | Cross sectional |
| 31 | Ageism in first episode psychosis | Mitford E, Reay R, McCabe K, Paxton R, Turkington D | 2010 | International Journal of Geriatric pPychiatry. 2010 Nov 1;25(11):1112-8. | UK | Psychosis | Retrospective |
| 32 | Equity of use of specialist palliative care by age: Cross-sectional study of lung cancer patients | Burt J, Plant H, Omar R, Raine R | 2010 | Palliative Medicine. 2010 Apr 15. | UK | Lung cancer | Cross sectional |
| 33 | Underutilization of parathyroidectomy in elderly patients with primary hyperparathyroidism | Wu B, Haigh P, Hwang R, Ituarte P, Liu I, Hahn T, and Yeh M | 2010 | The Journal of Clinical Endocrinology & Metabolism. 2010 Sep;95(9):4324-30. | USA | Primary hyperparathyroidism | Retrospective |
| 34 | Equality of access to “fast-track” stroke services in an older, socio-economically deprived Scottish population | Quinn T, Dawson J, Walters M | 2010 | Scottish Medical Journal. 2010 Aug 1;55(3):18. | UK | TIA | Prospective |
| 35 | Age and sex differences, and changing trends, in the use of evidence-based therapies in acute coronary syndromes: Perspectives from a multinational registry | Nguyen H, Goldberg R, Gore J, Fox K, Eagle K, Gurfinkel E, Spencer F, Reed G, Quill A, Anderson F | 2010 | Coronary Artery Disease. 2010 Sep 1;21(6):336-44. | USA | Acute coronary syndrome | Cross sectional |
| 36 | Age and rural residence effects on accessing colorectal cancer treatments: A registry study | Sankaranarayanan J, Watanabe-Galloway S, Sun J, Qiu F, Boilesen E, Thorson A | 2010 | The American Journal of Managed Care. 2010 Apr;16(4):265-73. | USA | Colorectal cancer | Retrospective |
| 37 | Age disparities in stroke quality of care and delivery of health services | Saposnik G, Black S, Hakim A, Fang J, Tu J, Kapral M | 2009 | Stroke. 2009 Oct 1;40(10):3328-35. | Canada | Stroke | Prospective |
| 38 | Mental health services use: Baltimore epidemiologic catchment area follow-up | Bogner H, de Vries H, Maulik P, Unützer J | 2009 | The American Journal of Geriatric Psychiatry. 2009 Aug 31;17(8):706-15. | USA | Any mental health condition | Prospective |
| 39 | Cancer pain management at home (I): do older patients experience less effective management than younger patients? | Bennett M, Closs S, Chatwin J | 2009 | Supportive Care in Cancer. 2009 Jul 1;17(7):787-92. | UK | Cancer | Cross sectional |
| 40 | Public mental health care utilization by older adults | Karlin B, Norris M | 2006 | Administration and Policy in Mental Health and Mental Health Services Research. 2006 Nov 1;33(6):730-6. | USA | Mental health care needs | Retrospective |
| 41 | Factors associated with the utilization and costs of health and social services in frail elderly patients | Kehusmaa S, Autti-Rämö I, Helenius H, Hinkka K, Valaste M, Rissanen P | 2012 | BMC Health Services research. 2012 Jul 19;12(1):1. | Finland | Frail elderly | Retrospective |
| 42 | Access to the kidney transplant wait list | Kiberd B, Boudreault J, Bhan V, Panek R | 2010 | American Journal of Transplantation. 2006 Nov 1;6(11):2714-20. | Canada | End-stage renal disease | Prospective |
| 43 | Equity of access to primary care among older adults in Incheon, South Korea | Park J | 2012 | Asia-Pacific Journal of Public Health. 2012 Nov 1;24(6):953-60. | Korea | Any primary health need | Cross sectional |
| 44 | Equity of access to long-term care among the American elderly | Park J | 2003 | Journal of Public Health. 2003 Jun 1;11(2):121-30. | USA | Long term care need | Cross sectional |
| 45 | Identification and documentation of persons being in palliative phase regardless of age, diagnosis and places of care, and their use of a sitting service at the end of life | Wallerstedt B, Sahlberg-Blom E, Benzein E, Andershed B | 2012 | Scandinavian Journal of Caring Sciences. 2012 Sep 1;26(3):561-8. | Norway | Palliative | Retrospective |
| 46 | Forgone health care due to cost among older adults in European countries and in Israel | Litwin H, Sapir E | 2009 | European Journal of Ageing. 2009 Sep 1;6(3):167-76. | Israel | Any healthcare needs | Cross sectional |
| 47 | Factors associated with self-reported use of oral health services among older Melbournians | Mariño R, Browning C, Kendig H | 2007 | Australasian Journal on Ageing. 2007 Sep 1;26(3):141-4. | Australia | Any dental | Cross sectional |
| 48 | Patient views of social service provision for older people with advanced heart failure | Gott M, Barnes S, Payne S, Parker C, Seamark D, Gariballa S, Small N | 2007 | Health & Social Care in the Community. 2007 Jul 1;15(4):333-42. | UK | Heart failure | Prospective |
| 49 | Underuse of inhaled steroid therapy in elderly patients with asthma | Sin D, Tu J | 2001 | CHEST Journal. 2001 Mar 1;119(3):720-5. | Canada | Asthma | Retrospective |
