# Supplementary material for: Identifying inequitable healthcare in older people: systematic review of current research practice
Source: Int J Equity Health. 2017 Jul 11;16:123. doi: 10.1186/s12939-017-0605-z (PMC5505033; doi:10.1186/s12939-017-0605-z)
Supplement: Supplementary file 5 — Studies reporting higher receipt-need ratio among younger than older groups in at least one outcome (N = 36) (DOCX 26 kb) [file 12939_2017_605_MOESM5_ESM.docx]

**Additional File 5: Studies reporting higher receipt-need ratio among younger than older groups in at least one outcome (N=36)**

| # | Authors | Date | Contra-indications mentioned  yes/no | Effectiveness at older age mentioned yes/no | Patient preference mentioned yes/no | Conclusion | Illustrative quote |
| --- | --- | --- | --- | --- | --- | --- | --- |
| 1 | Jin X, et al | 2014 | No | Yes | No | None beyond report of age-related patterns | *" DMARD use in elderly RA patients was noticeably low, ..., clinicians may need to pay more attention to elderly RA patients" (p210)* |
| 2 | Hermosillo-Rodriguez J, et al. | 2013 | Yes | Yes | Yes | Warranted difference suggested | *“…none of the older patients receiving adjuvant chemotherapy in this population were referred to skilled nursing facilities in the six months following initiation of therapy. This finding further supports the potential strength of oncologists' abilities in selecting appropriate chemotherapy recipients.” “As people age, overall survival tends to become a less important health goal compared to quality of life and the burden of morbidity. These patient-centred health goals have an impact on patients' preferences for adjuvant chemotherapy"* p100 |
| 3 | Gottlieb S, et al. | 2011 | Yes | Yes | No | Unwarranted difference suggested | *“Since treatment guidelines are age neutral, adherence to these guidelines may prevent bias, and inequality of treatment of the elderly.”* P123 |
| 4 | Reuber M, et al. | 2010 | No | No | No | Unwarranted difference concluded | “Our findings provide clear evidence of inequity of access to specialist services across the age range that our adult service is intended to serve.” “…the most likely explanation for our findings is that general practitioners or emergency room physicians are less likely to refer older patients to the specialist epilepsy service.”p2343 |
| 5 | Forman D, et al. | 2009 | Yes | Yes | No | Unwarranted difference suggested | *“Get With The Guidelines–Heart Failure data demonstrate that guidelines recommended therapies are frequently utilized for older patients with HF, including patients >85 years old. Nonetheless, age-related differences in therapy persist, suggesting that opportunities to improve care still remain”*p1010 |
| 6 | Jung B, et al. | 2009 | Yes | Yes | No | None beyond report of age-related patterns | *"Treatment of rectal cancer is influenced by patient's age. Future studies should include younger and older patients alike to reveal whether or not age-related differences are*  *purposive"* p1 |
| 7 | Burge F, et al. | 2008 | No | No | Yes | None beyond report of age-related patterns | *“In the future we require finer definitions of "need" before statements can be made beyond inequalities to that of equity. We also require information about patient preferences"* p1210 |
| 8 | Neighbors H, et al. | 2008 | No | No | No | Unwarranted difference suggested | *"These findings suggest that ... not everyone who needs care is receiving it"p953 "raises questions about the types and adequacy of treatment older African Americans are receiving for mental disorders"* p953 |
| 10 | Prina A, et al. | 2014 | No | Yes | No | Unwarranted difference concluded | *“Access to the IAPT services for older adults is lower than expected, given household survey estimates of the prevalence of depression. It probably reflects how these services were set up but, given the outcome data presented above, this limited access needs attention in order to address age discrimination in service access.”p81* |
| 11 | Jokela M, et al. | 2013 | No | No | No | Unwarranted difference suggested | *"Ageing is also associated with an increasing discrepancy between prevalence of mental*  *disorders and provision of treatment, as indicated by lower use of psychotherapy in older individuals."* p2037 |
| 12 | Crabb R, Hunsley J | 2009 | No | Yes | No | Unwarranted difference concluded | “…the present study provides compelling evidence that mental health services are particularly underutilized by depressed older adults.” P299 |
| 13 | Chaix B, et al. | 2005 | No | No | No | Unwarranted difference suggested | *"Our study suggests that the elderly combining a personal disadvantage (impaired mobility) with a residential disadvantage (living in an underserved area) have a dramatically reduced access to primary care."* p287 |
| 14 | Keating N, et al. | 2006 | Yes | No | Yes | Unwarranted difference suggested | *"Many elderly breast cancer survivors do not undergo annual*  *Surveillance mammography.... These disparities are not explained by access to follow-up care. New strategies are needed to promote surveillance mammography and decrease variations based on nonclinical factors that are likely unrelated to appropriateness of medical care" p92* |
| 15 | Bhalla A, et al. | 2004 | Yes | No | Yes | Unwarranted difference suggested | *“This is suggestive of possible age discrimination in certain centres ..In order for stroke care to be effective, ward settings fulfilling organised specialist stroke care should ensure they deliver on all components of this to all patients, young and old”p622-3* |
| 16 | Hunt K, et al. | 2014 | Yes | No | Yes | Unwarranted difference suggested | *“A degree of age-associated disparity in the recording and realization of preference for place of death...Complex biopsychosocial needs bring challenges to the provision of high-quality end-of-life care for the oldest old, and our findings support the notion that the oldest old are the ‘disadvantaged dying... Relatives of the oldest old reported poorer quality care in the last two days and reduced choice in*  *their care"* p180-1 |
| 18 | Joerger M, et al. | 2013 | Yes | Yes | Yes | Unwarranted difference concluded | *"The present study provides evidence for incomplete staging and a substantial underuse of BCS versus MX, post-BCS radiotherapy*  *and adjuvant endocrine treatment in elderly patients with early BC" p45 "There is a need for improved management of early BC in the elderly even in a system with universal access to health care services" p46* |
| 19 | Steele S, et al. | 2014 | Yes | No | Yes | Unwarranted difference suggested | *“under-treatment in older patients” “further research may elucidate the cause of the disparity in treatment usage among differing age groups"* p 308 |
| 20 | Nauta S, et al. | 2013 | Yes | Yes | No | Unwarranted difference concluded | *“Older patients with an MI remained less likely to receive evidence-based care during 24 years of observation. .. The application of proven MI therapies to appropriate patients regardless of age may even further improve these outcomes”*p693 |
| 21 | Austin S, et al. | 2013 | Yes | Yes | No | Unwarranted difference concluded | *“This study indicates that although the benefits associated with the physical activity is well recognised, there is age bias in physicians' recommendations for physical activity"* p222 *“The findings imply an issue of poor quality of care provided."*p229 |
| 22 | Von Heymann-Horan A, et al. | 2013 | No | No | Yes | None beyond report of age-related patterns | *"The effect of age may be based on several mechanisms." p361* |
| 25 | Palmcrantz S, et al. | 2012 | No | Yes | Yes | None beyond report of age-related patterns | *"Younger individuals received more care and rehabilitation, which indicates structural inequality in the provision of health care resources. However, as no difference in self-perceived global recovery was found between the groups; the disparity in the provision of health care may also be a consequence of greatest support being given to those in greatest need” p29* |
| 26 | Bajorek B, Ren S | 2012 | Yes | Yes | Yes | Unwarranted difference concluded | *"Clinicians appear to be taking evidence-based considerations regarding contraindications and stroke risk into account*  *more so than before, with less of the previously reported ‘ageism’ in their decision-making....around half of eligible patients 80 years or older did not receive warfarin therapy. It is a concern that warfarin underuse continues to be apparent in these most stroke prone patients who would benefit the most from receiving it"* p 94 |
| 27 | Ryb G, Dischinger P | 2011 | No | Yes | No | Unwarranted difference concluded | *"In contrast to the American College of Surgeons triaging recommendations, injured MVC occupants older than 60 years are less likely to be transported to a trauma centre than their younger counterparts ...further studies should establish whether ... is a function of geographical factors, emergency medical services unconscious bias or other factors"* p 742 |
| 28 | Singh P, et al. | 2011 | Yes | Yes | No | Unwarranted difference concluded | *“Our results indicate that antithrombotic therapy, particularly warfarin, is underused in ACF-dwelling [aged care facility] elderly patients who are eligible for treatment.”*p166 |
| 29 | Cooper C, et al. | 2010 | Yes | Yes | No | Unwarranted difference concluded | “Older people are less likely to receive evidence-based treatment for CMD. Managers and clinicians should prioritise reducing this inequality.” p96 |
| 30 | Knauer S, et al. | 2010 | Yes | Yes | Yes | None beyond report of age-related patterns | *" Further research should examine the factors underlying age-related differences in provider and treatment use to determine whether older North*  *Carolinians are receiving appropriate care for the treatment of chronic LBP"* p1231 |
| 31 | Mitford E, et al. | 2010 | No | Yes | No | Unwarranted difference concluded | *"There is a substantial proportion of older people with first episode psychosis... There are large gaps in services for this group who often do not have the same access to those offered to younger people...Ageism exists in all forms; the elderly are doubly disadvantaged in view of their age and mental illness. In view of the Age Discrimination Act (2006) the elderly functionally ill group of patients should be entitled to the same level of care and equal access to services as younger people. More attention and interventions need to be focused on this overlooked group" p1112* |
| 33 | Wu B, et al. | 2010 | Yes | Yes | Yes | Unwarranted difference concluded | *"PHPT is undertreated in the elderly"* p 4324 *“Our data demonstrate an inverse relationship between age and likelihood of PTx [parathyroidectomy]. This effect is independent of comorbidity, gender, race, clinical and biochemical disease severity, and objective standards regarding appropriateness of surgical care. In other words, older patients, as a group, undergo fewer parathyroid operations than their younger counterparts, apparently based in large part on age alone”*p4328 |
| 35 | Nguyen H, et al. | 2010 | Yes | Yes | Yes | Unwarranted difference suggested | *"Underlying reasons for differences in the use of effective treatment modalities by age and sex need to be more fully elucidated to narrow the current treatment disparities."* p344 |
| 36 | Sankaranarayanan J, et al. | 2010 | Yes | Yes | Yes | None beyond report of age-related patterns | *"Further investigation is needed to understand .. the decreased likelihood of all cancer treatment types among older patients with*  *colon cancer and with rectal cancer"* p272 |
| 38 | Bogner H, et al. | 2009 | No | No | Yes | Unwarranted difference concluded | "Older adults continue to underutilize speciality mental health services compared with middle-aged adults .... Effective ways of facilitating entering into the speciality mental health services sector including community education and outreach should be examined"p712-3 |
| 40 | Karlin B, Norris M | 2006 | No | Yes | No | Unwarranted difference suggested | *“Results are surprisingly consistent with previous findings of substantial underutilization of mental health care by older adults... effective methods of facilitating older adults’ direct and indirect entry into the*  *specialty mental health service sector (e.g., physician education, community education and outreach)*  *should be examined."* p 734-5 |
| 42 | Kiberd B, et al. | 2010 | Yes | Yes | No | None beyond report of age-related patterns | *“About 20% have neither contraindication nor are referred. This group is elderly and the majority has comorbidities that impact on life expectancy. Clinicians are probably incorporating these factors into their decision-making process when considering referral”*p2714 |
| 43 | Park J | 2012 | No | No | No | Unwarranted difference concluded | *“The present study examines the extent to which equity in the use of physician services for the elderly has been achieved in Incheon, Korea....The results indicate that a universal health insurance system has not yielded a fully equitable distribution of services" p953* |
| 47 | Mariño R, et al. | 2007 | No | No | No | Unwarranted difference suggested | *“Regular dental visits become even more relevant when considering older populations.... Currently, more than ever before, older people have to compete for access with other age groups of cardholders on a ‘first-come first-served’ basis.”*p143 |
| 49 | Sin D, Tu J | 2001 | Yes | Yes | No | Unwarranted difference concluded | *“Although neither the American nor Canadian consensus guideline statements has indicated that the use of inhaled steroid therapy should be modified by age or comorbidities, our findings are consistent with previous reports that asthma is underdiagnosed and undertreated in the elderly population”*p723 |
